# Zbtb1 controls NKp46^+^ ROR-gamma-T^+^ innate lymphoid cell (ILC3) development

**DOI:** 10.18632/oncotarget.19645

**Published:** 2017-07-27

**Authors:** Ying Lu, Xianyu Zhang, Nicolas Bouladoux, Saransh Neel Kaul, Kangxin Jin, Derek Sant’Angelo, Yasmine Belkaid, Damian Kovalovsky

**Affiliations:** ^1^ Experimental Immunology Branch, NCI, NIH, Bethesda, MD, USA; ^2^ Mucosal Immunology Section, Laboratory of Parasitic Diseases, NIAID, NIH, Bethesda, MD, USA; ^3^ University of Maryland, College Park, MD, USA; ^4^ Zhongshan Ophthalmic Center, State Key Laboratory for Ophthalmic Researches, Sun Yat-Sen University, Guangzhou, Guangdong, China; ^5^ Cancer Metabolism and Growth Program, Rutgers, Child Health Institute of New Jersey, New Brunswick, NJ, USA; ^6^ Experimental Transplantation and Immunology Branch, NCI, NIH, Bethesda, MD, USA

**Keywords:** innate lymphoid cells, ILC, ILC3, Zbtb1, IFN-γ, Immunology and Microbiology Section, Immune response, Immunity

## Abstract

Innate lymphoid cells (ILCs) play a central role conferring protection at the mucosal frontier. In this study, we have identified a requirement of the transcription factor Zbtb1 for the development of RORγt^+^ ILCs (ILC3s). Zbtb1-deficient mice lacked NKp46^+^ ILC3 cells in the lamina propria of the small and large intestine. This requirement of Zbtb1 was cell intrinsic, as NKp46^+^ ILC3s were not generated from Zbtb1-deficient progenitors in bone marrow chimeras and Zbtb1-deficient RORγt^+^ CCR6^−^NKp46^−^ ILC3s didn't generate NKp46^+^ ILC3s in co-cultures with OP9-DL1 stroma. In correlation with this impairment, Zbtb1-deficient ILC3 cells failed to upregulate T-bet expression, and to acquire IFN-γ production characteristic of NKp46^+^ cells. Finally, absence of NKp46^+^ILC3 cells combined with the absence of T-cells in Zbtb1-deficient mice, led to a transient susceptibility to *C. rodentium* infections. Altogether, these results establish that Zbtb1 is essential for the development of NKp46^+^ ILC3 cells.

## INTRODUCTION

The intestinal mucosa is a permeable barrier that allows absorption of nutrients while preventing invasion of pathogenic bacteria. Innate lymphoid cells (ILCs) are present at mucosal surfaces and represent a first line of defense against bacterial infections [1].

ILCs represent a family of innate effectors that lack expression of lineage-specific markers corresponding to T, B and myeloid cells. Although they develop from a common lymphoid progenitor (CLP, Lin-Sca-1^low^c-kit^low^CD127^+^) and express CD127 (IL-7Rα) as lymphoid cells, they lack rearranged antigen receptors such as the BCR in B-cells and TCR in T-cells [2, 3].

Distinct cytokine patterns and lineage-specific transcription master regulators identify different ILC effector populations, which highly resemble CD4^+^ helper T cell subsets. Group 1 ILCs (ILC1s) produce IFN-γ and express the transcription factor T-bet. ILC2s secrete IL-13 and express the transcription factor Gata3 and IL22-producing ILC3s express Rorγt [4, 5]. Rorγt^+^ ILC3 cells are a heterogeneous population that is particularly abundant at mucosa sites. They can be divided into three main subsets based on their role during embryogenesis and their cell-surface expression of the natural cytotoxicity receptor NKp46 [6]. NKp46-CCR6+ILC3s are lymphoid tissue-inducer cells (LTi) that were originally identified in the fetus and are required for the development of lymph nodes and Peyer's patches [7, 8]. NKp46^−^CCR6^−^ILC3s are the precursors of NKp46^+^CCR6^−^ILC3s cells which develop after birth [6]. After birth, a subset of CCR6^−^ ILC3s upregulate T-bet in response to the microbiota and Notch signals, express NKp46, high levels of the IL-12 receptor, and acquire IFN-γ secretion [6, 9]. Postnatal NKp46^+^ ILC3s were shown to have a specific role in preventing inflammation and ulceration of the Caecum after *C. rodentium* infections in immune competent mice and play a redundant role with other ILC3 subsets and T-cells in protecting the gastrointestinal tract [10–12] . IL-22-deficient mice [13] and genetic disruption of ILC3 development, as it occurs in mice deficient for T-bet (Tbx21-deficient), TCF-1-deficient or AhR-deficient mice are highly susceptible to *C. rodentium* infections [14–16].

The transcription factors in the BTB-ZF (Broad complex, tramtrack, and Bric a brac-zinc finger) family are critically involved in lymphoid commitment and development. Zbtb16 (PLZF), a transcriptional regulator also linked to the function of natural killer T cells and IL-17^+^γδT-cells [17, 18], marks a subset of ILC lineage-specific progenitor cells. These cells express the integrin α4β7 and can give rise to helper ILCs, excluding LTi cells and natural killer cells [19]. Zbtb1 has been recently shown to play an important role in the development of lymphocytes. A point mutation in the BTB domain of Zbtb1 (ScanT mutant) and Zbtb1 deficiency lead to complete absence of T lymphocytes [20]. Development of other peripheral lymphoid lineages including B cells and NK cells as well as myeloid cells are also partially impaired, and their development is severely compromised under competitive conditions in mixed bone marrow chimeras [20–22]. It was recently identified that Zbtb1 prevents DNA damage in cell lines by initiating translesion DNA synthesis in response to replication stress and absence of Zbtb1 leads to increased DNA damage, and activation of p53-dependent apoptosis in immune progenitors, which impacts the generation of lymphoid and myeloid lineages [23, 24].

In this report, we have identified a requirement of Zbtb1 for the normal development of ILCs. Despite the ubiquitous expression of Zbtb1 in different ILC subsets, Zbtb1 greatly impacted the generation of NKp46^+^ILC3s, while normal numbers of ILC1 and ILC2 subsets were found in the intestine of ScanT mice. This requirement of Zbtb1 was cell intrinsic and not related to p53-dependent apoptosis of NKp46-ILC3 progenitors. Absence of NKp46^+^ILC3s cells in ScanT mice correlated with a transient susceptibility to *C.rodentium* infections underscoring a specific function of this subset in protective immunity. In summary, our results identify Zbtb1 as a key transcription factor required for the generation of NKp46^+^ ILC3s cells.

## RESULTS

### Zbtb1 is expressed in ILC progenitors and mature ILC lineages

**Figure 1:**
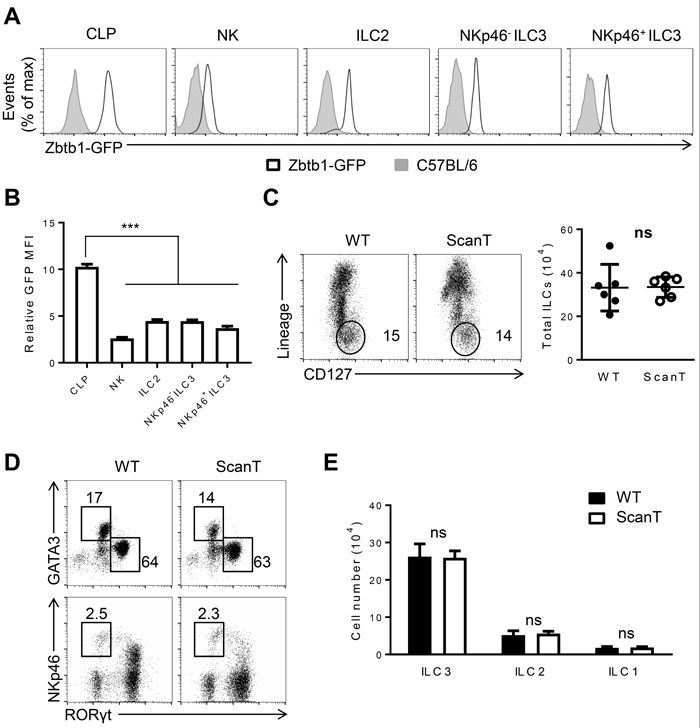
Zbtb1 is expressed in ILC progenitors and mature ILC lineages **A.** Flow cytometry of GFP positive cells among the CLPs from BM, NK, ILC2, NKp46-ILC3 and NKp46+ ILC3 cells from siLP of C57BL/6 and Zbtb1-GFP reporter mice. **B.** Relative GFP Mean fluorescence intensity (MFI) was calculated in Zbtb1-GFP mice relative to C57BL/6 mice as represented in **A.**. **C.** Flow cytometry of siLP cells from WT and ScanT mice, stained with CD127 and Lineage markers (TCRβ, TCRδ, CD19, CD11c, CD11b, Gr-1, Ter119,) among live CD45.2^+^ lymphocytes. Number of total ILCs among lymphocytes from siLP of WT and ScanT mice is represented in the right. **D.** Flow cytometry of ILCs from siLP stained for GATA3, Rorγt and NKp46. **E.** Numbers of ILC1, ILC2 and ILC3 cells as shown in **D.** in the siLP of WT and ScanT mice. All graphs display means ± SD. Results are representative of at least three independent experiments with six to twelve mice in each experimental group. Numbers adjacent to outlined areas indicate the percentage of events within the gate. * *P* < 0.05, ** *P* < 0.01, *** *P* < 0.001 and ^ns^ *P* > 0.05 (two-tailed unpaired Student's *t*-test).

Zbtb1 is required for the development of peripheral lymphocytes [20, 21]. As innate lymphoid cells (ILCs) share the same precursors with peripheral lymphocytes, we were interested to evaluate how Zbtb1 affected the development of ILC lineages. To this end, we first examined the expression level of Zbtb1 in common lymphoid progenitors (CLPs, Lin^−^Sca-1^low^c-kit^low^CD127^+^) from bone marrow as well as ILC lineages in the lamina propria of the small intestine (siLP). By analyzing GFP levels in the BAC transgenic Zbtb1-GFP reporter mice (ZEG), we identified that CLPs are already Zbtb1-expressing cells and ILC lineages in the siLP express Zbtb1 at lower levels (Figure 1A and 1B). To analyze the impact of Zbtb1 to ILC development, we used the ScanT strain in which a point mutation in the Zbtb1C74R severely disrupts T-cell development due to absence of Zbtb1 protein [20, 24]. Surprisingly, contrarily to the profound requirement of Zbtb1 for adaptive lymphoid development, Zbtb1-deficiency didn't affect the generation of ILC cells, wild type and ScanT mice had a similar percentage and cell number of ILCs in the siLP identified as Lin^−^CD127^+^ cells (Figure 1C). The generation of ILC2 (GATA3^+^), ILC3 (Rorγt^+^) and ILC1 (Rorγt^−^ GATA3^−^ NKp46^+^) subsets was not significantly altered in ScanT mice either (Figure 1D, 1E).

### Zbtb1 is required for the generation of NKp46^+^ ILC3 cells

**Figure 2:**
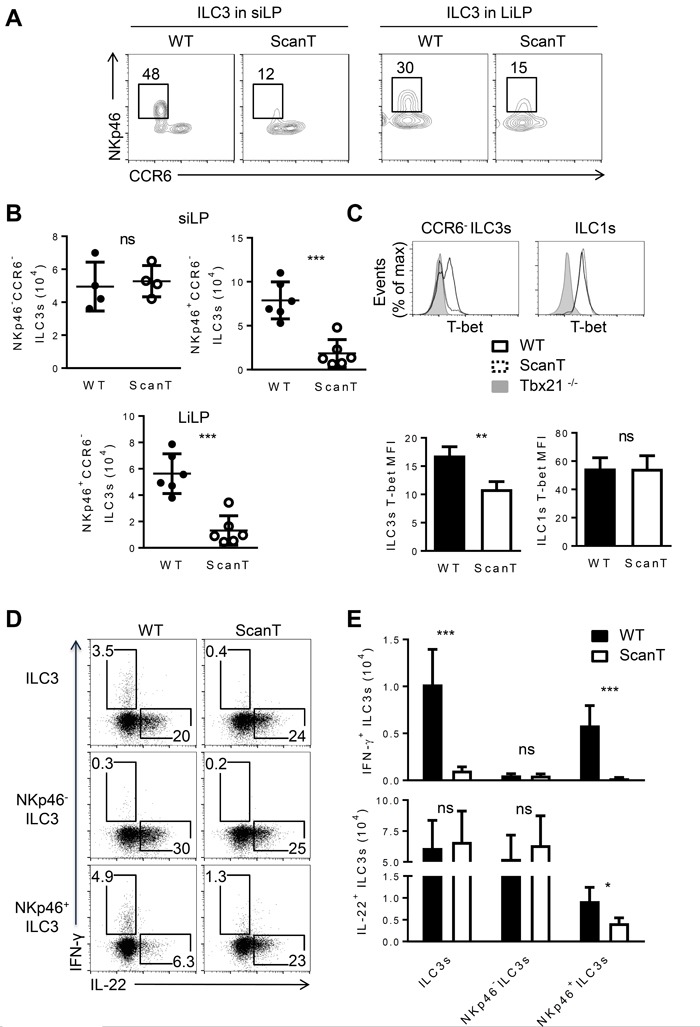
Zbtb1 is required for the normal development of NKp46^+^CCR6^−^ ILC3s **A.** Flow cytometry of Rorγt^+^ ILC3s from siLP (left) and LiLP (right) from WT and ScanT mice. **B.** Numbers of the indicated ILC3 subsets from the siLP and LiLP from WT and scanT mice. **C.** FACs analysis of T-bet levels in CCR6^−^ ILC3 and ILC1 cells (top) and quantitation of T-bet MFI (bottom). **D.** Intracellular IL-22 and IFN-γ levels in NKp46^−^, NKp46^+^ and total ILC3s from siLP after stimulation with phorbol 12-myristate 13-acetate (PMA) and ionomycin in the presence of brefeldin A for 3 hours. **E.** Absolute numbers of IFN-γ^+^ and IL-22^+^ cells stained as **D.**. All graphs display means ± SD. Results are representative of at least three independent experiments with three mice in each experimental group. Numbers adjacent to outlined areas indicate the percentage of events within the gate. * *P* < 0.05, ** *P* < 0.01, *** *P* < 0.001 and ^ns^ *P* > 0.05 (two-tailed unpaired Student's *t*-test).

Since Rorγt^+^ ILC3 cells can be further categorized into several subsets with phenotypical and functional markers, we examined if Zbtb1 differentially affected the generation of ILC3 subsets. CCR6^+^NKp46^−^ ILC3 cells correspond to lymphoid tissue inducer-like (LTi) cells [6]. CCR6^−^ILC3 cells correspond to an independent lineage that develops postnatally and acquire NKp46 expression and IFN-γ secretion after extensive proliferation [25]. We observed that Zbtb1-deficiency severely affected the generation of NKp46^+^CCR6^−^ ILC3 cells as both the percentage and absolute number of NKp46^+^CCR6^−^ ILC3s were significantly decreased in the small and large intestine of ScanT mice (Figure 2A and 2B). This correlated with an increase of CCR6^+^ILC3 cells in ScanT mice (Supplementary Figure 1). In correlation with deficient generation of NKp46^+^ILC3 cells, ScanT mice had reduced T-bet levels in CCR6^−^ILC3 cells, but the ScanT mutation didn't affect T-bet levels in ILC1 cells (Figure 2C), suggesting that Zbtb1 controls a developmental transition in ILC3s but does not directly control T-bet levels. This was also evident as the few NKp46^+^ILC3 cells generated in ScanT mice presented normal T-bet levels (Supplementary Figure 2). We then analyzed if Zbtb1 affected cytokine secretion in ILC3 cells. We observed that Zbtb1-deficiency didn't affect IL-22 secretion in total ILC3 cells after PMA/Ionomycin stimulation *in vitro* (Figure 2D and 2E) or after stimulation of cells with IL-23, which is a more physiological stimulus. We have also observed that an increased proportion of ScanT NKp46^+^ ILC3 cells were IL22 producing cells (Supplementary Figure 3). However, Zbtb1 deficiency led to blunted IFN-γ in total ILC3s and in NKp46^+^ILC3 cells (Figure 2D and 2E) in correlation with their inability to upregulate T-bet in ScanT ILC3s.

### The requirement of Zbtb1 for the generation of NKp46^+^ILC3s is cell-intrinsic

**Figure 3:**
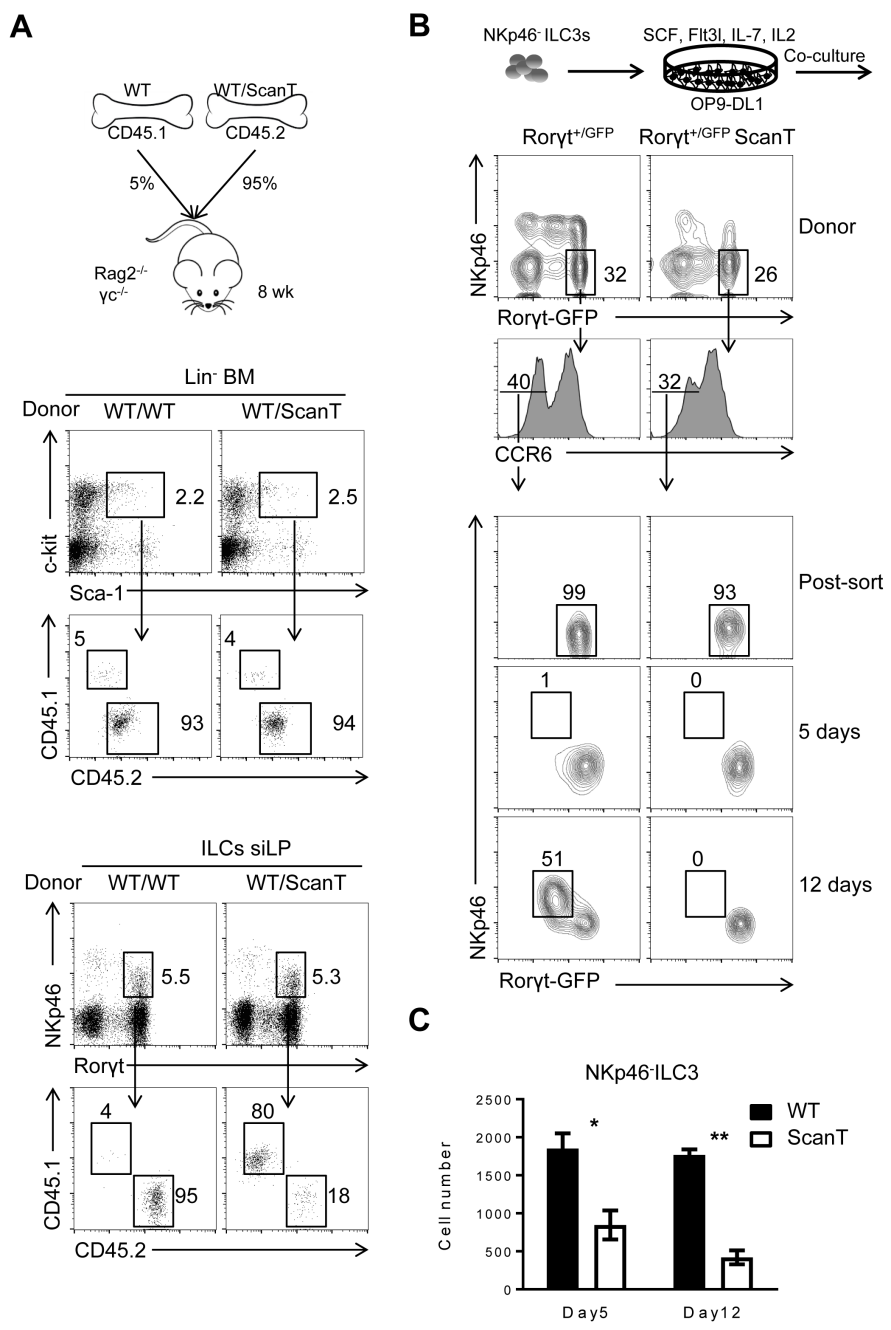
Cell intrinsic requirement of Zbtb1 for the generation of NKp46^+^ILC3s **A.** Diagram representing the experimental strategy of Bone Marrow Chimeras, CD45.1^+^ cells correspond to cells derived from wild type bone marrow in competition with CD45.2^+^ cells corresponding to either wild type or ScanT bone marrow (top); Analysis of Lin^−^Sca1^+^cKit^+^ progenitor cells in bone marrow (middle); Analysis of Lin^−^CD127^+^ Rorγt^+^ NKp46^+^ILC cells in siLP. **B.** Experimental strategy of *in vitro* differentiation of CCR6^−^NKp46^−^ILC3s sorted from the siLP of Rorγt^+^/^GFP^ and Rorγt^+^/^GFP^ ScanT compound mice and cultured on OP9-DL1 stromal cells for 5 or 12 days in the presence of SCF, Flt3L, IL-7 and IL-2. Flow cytometry of cultured cells (gated on Lin^−^ cells) from siLP of indicated mice. **C.** Absolute number of cultured cells (Lin^−^) obtained after culture in **B.**. Results are representative of at least three independent experiments with three mice in each experimental group. Numbers adjacent to outlined areas indicate the percentage of events within the gate. * *P* < 0.05 and ** *P* < 0.01 (two-tailed unpaired Student's *t*-test).

**Figure 4:**
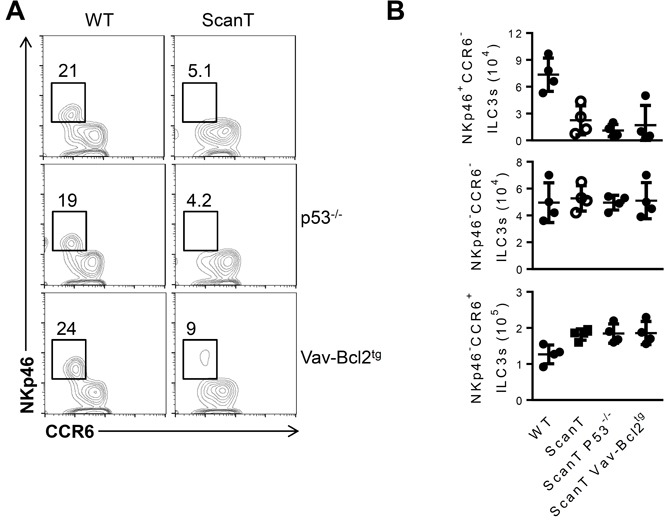
Transgenic Bcl2 expression or p53-deficiency does not revert the impaired generation of NKp46^+^ ILC3 cells in ScanT mice **A.** Analysis of ILC3 subsets in the siLP from the indicated mice, gated as Lin^−^CD127^+^Rorgt^+^as in Figure 1D in the indicated compound mice (wild type, ScanT, p53^−/−^, p53^−/−^ScanT, Vav-Bcl2^tg^ and Vav-Bcl2^tg^ScanT). Numbers represent the frequency of cells within the gate. **B.** Cell numbers of the indicated subset and strain of mice. Data is representative of at least three independent mice analyzed per group.

To interrogate if the requirement of Zbtb1 for the generation of NKp46^+^ILC3 cells was cell-intrinsic, we generated mixed bone marrow (BM) chimeras in which we transplanted Rag2^−/−^γc^−/−^ recipient mice with a 1:20 ratio of wild type/wild type or wild type/ScanT BM (Figure 3A). We used this proportion as an attempt to compensate for the increased p53-dependent apoptosis of ScanT immune progenitors, which affects development of all immune cell lineages [24]. 8 weeks post-transplantation we analyzed the generation of Lin^−^Sca1^−^cKit^−^ (LSK) progenitors in bone marrow and corroborated a similar contribution between wild type and ScanT cells as previously reported [24]. Although NKp46^+^ILC3 cells were generated from donor progenitors, these cells were derived from wild type and not from ScanT progenitors (Figure 3A; NKp46+ILC3 WT = 82± 1.764 *vs* ScanT = 16.67 ± 2.404, *n* = 3, *p* < 0.001 ), demonstrating a cell-intrinsic requirement of Zbtb1 for the generation of this subset. However, as predicted by the requirement of Zbtb1 in hematopoietic progenitors, the generation of NKp46^−^ILC3 cells from ScanT progenitors was also impaired in competitive mixed bone marrow chimeras (Supplementary Figure 4). NKp46^+^ILC3 cells can develop from NKp46^−^ILC3 progenitors by co-culture with stroma cells expressing the Notch ligand DL1 (OP9-DL1) [6, 14]. To test if Zbtb1-deficiency affected the generation of NKp46^+^ILC3 cells from NKp46^−^ILC3 progenitors, we generated compound Rorγt^+/GFP^ ScanT mice, sorted NKp46^−^ ILC3s from Rorγt^+/GFP^ and Rorγt^+/GFP^ ScanT mice and co-cultured them on OP9-DL1 stromal cells in the presence of SCF, Flt3L, IL-7 and IL-2 cytokines. After 12 days, Rorγt^+/GFP^ NKp46^−^ ILC3s generated NKp46^+^ ILC3s, whereas Rorγt^+/GFP^ ScanT NKp46^−^ ILC3s did not generate this subset (Figure 3B; NKp46+ILC3 WT = 47 ± 3.512 *vs* ScanT = 0, *n* = 3). ScanT cell numbers were significantly reduced after culture, in correlation with increased sensitivity to replication stress and apoptosis of immune cells from this strain when induced to proliferate [23, 24] (Figure 3C). Therefore, NKp46^−^ ILC3s were not able to develop into NKp46^+^ cells in response to Notch signals when lacking functional Zbtb1. To test if this developmental defect was due to increased p53-dependent apoptosis, we crossed ScanT mice with Vav-Bcl2 transgenic mice as well as p53^−/−^ mice, which successfully reverted the apoptosis and impaired lymphoid and myeloid development in ScanT mice [24], and analyzed if NKp46^+^ILC3 cells were present in the siLP of compound mice. Surprisingly, neither Bcl2 overexpression nor p53 deficiency could rescue the generation of NKp46^+^ ILC3s in ScanT mice (Figure 4A and 4B), suggesting that absence of NKp46^+^ILC3 cells is independent of replication stress and the p53-dependent apoptosis that affects the generation of other immune lineages [23, 24].

### Zbtb1 contributes to host defense against *C. rodentium* infections

**Figure 5:**
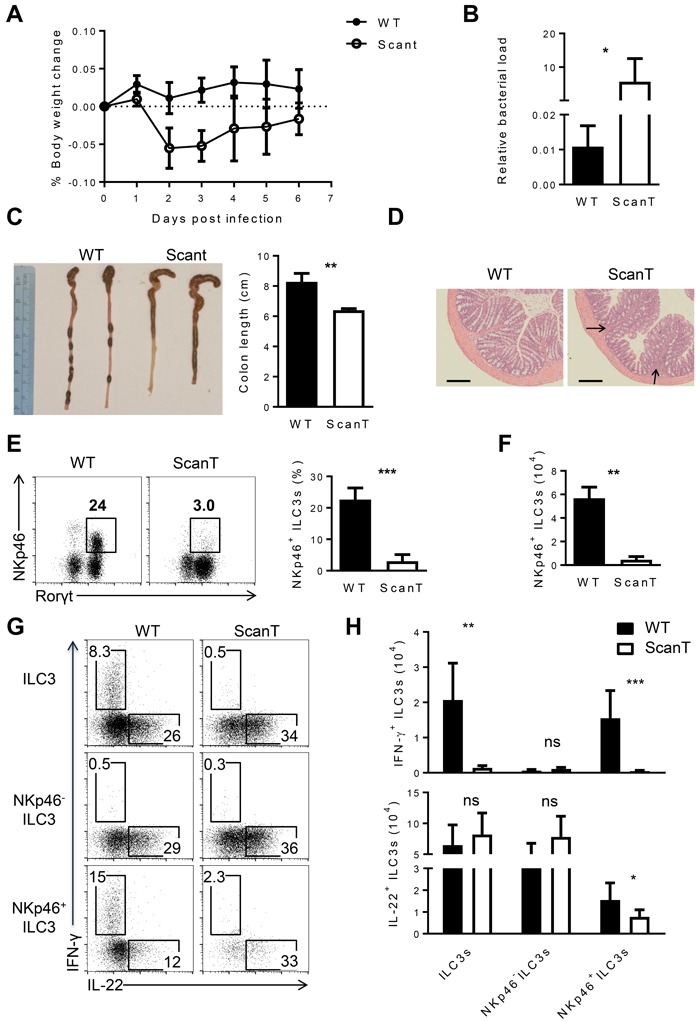
Zbtb1 is critical for host defense against *C. rodentium* **A.** Weight loss in WT and Scant mice after *C. rodentium* infection. **B.** Real-time PCR of *espB* gene relative to *Actb* in the feces from WT and Scant mice 4 days after *C. rodentium* infection. **C.** The length of colon and **D.** histological analysis of colons in WT and Scant mice 6 days after *C. rodentium* infection. Arrows show inflammatory infiltration. Scale bars, 100 μM. **E.** The percentage of NKp46^+^ ILC3 cells and **F.** the absolute number of these cells derived from WT and Scant mice 4 days after *C. rodentium* infection. **G.** Intracellular IL-22 and IFN-γ expression in NKp46^−^, NKp46^+^ and total ILC3s from siLP after stimulation with PMA and ionomycin in the presence of brefeldin A for 3 hours. **H.** Absolute numbers of IFN-γ^+^ and IL-22^+^ cells stained as **G.**. All graphs display means ± SD. Results are representative of at least three independent experiments with three mice in each experimental group. Numbers adjacent to outlined areas indicate the percentage of events within the gate. * *P* < 0.05, ** *P* < 0.01, *** *P* < 0.001 and ^ns^ *P* > 0.05 (two-tailed unpaired Student's *t*-test).

To investigate if Zbtb1 was required for the function of ILC3 cells in protecting mice from bacterial infections, we evaluated if ScanT mice were more susceptible to *C. rodentium* infections. NKp46^+^ILC3 cells protect against *C.rodentium* infection in immunocompromised mice, lacking T-cells, but not in immunocompetent mice [12]. As ScanT mice are deficient of T cells, we analyzed the susceptibility of this strain to *C.rodentium* infection. We choose very early time points for this analysis as T-cells from wild type mice do not contribute to the pathology at the early stages [14]. At 2 and 3 days post infection, ScanT mice significantly lost more body weight than wild type mice, indicative of a deficient innate response to the pathogen (Figure 5A). This increased susceptibility was transient as body weight didn't continue to decrease at later time points, in agreement with possible compensatory mechanisms from other ILC3 subsets. Inefficient control of the bacterial infection was reflected by a significantly increased load of bacteria in the feces, measured as an increase of the *C. rodentium* DNA relative to mammalian DNA (*EspB/Actb*) (Figure 5B) and a significantly increased shortening of the colons (Figure 5C). Histological analysis of the colon revealed areas of immune cell infiltration in ScanT mice, but no overt inflammation or epithelial hyperplasia was observed (Figure 5D), indicative of a mild pathology.

To investigate if the deficiency of NKp46^+^ ILC3 cells was still observed in ScanT infected mice, we analyzed their presence in siLP 4 days after infection. Similarly to naïve mice, ScanT infected mice presented a significant reduction of NKp46^+^ ILC3 cells in siLP (Figure 5E and 5F). ILC3 cells from infected ScanT mice didn't secrete IFN-γ and showed a mild but significant decrease of IL-22-secreting cells within NKp46^+^ cells, although the number of IL-22-producing ILC3 cells was comparable between WT and ScanT mice (Figure 5G and 5H).

Altogether, we have identified a cell-intrinsic requirement of the transcription factor Zbtb1 for the generation of NKp46^+^ ILC3 cells. Despite expression of Zbtb1 in all ILC subsets in the intestinal mucosa, Zbtb1 was essential for the generation of NKp46^+^ILC3 cells and IFN-γ secretion by ILC3s. This phenotype correlated with increased susceptibility to *C.rodentium* infections in the T-cell deficient background of ScanT mice, underscoring a specific function of NKp46^+^ILC3 cells and IFN-γ in conferring innate protection at the mucosal barrier.

## DISCUSSION

In this study, we have uncovered an essential requirement of the transcription factor Zbtb1 for the generation of NKp46^+^ILC3 cells. ScanT (Zbtb1-deficient) mice lack T-cells and present a profound deficiency in the generation of lymphoid and myeloid immune cells [20–22, 24]. This phenotype is due to an inefficient replication stress response [23], which leads to increased DNA damage and activation of the p53-dependent apoptosis when immune progenitors undergo rapid proliferation. Interestingly, although transgenic Bcl2 expression and p53-deficiency recovered the generation of B-cells and myeloid cells in ScanT mice [24], it was not sufficient to recover the generation of NKp46^+^ILC3 cells, suggesting that Zbtb1 is exerting a specific function in the generation of this subset. Zbtb1-requirement was cell intrinsic as ScanT progenitors in bone marrow chimeras failed to generate NKp46^+^ILC3 cells, and we further delineated a specific requirement of Zbtb1 for the generation of NKp46^+^ILC3 cells from CCR6^−^NKp46^−^ILC3 progenitors in co-cultures with stroma cells expressing the Notch1 ligand OP9-Dl1, an *in vitro* developmental system that was previously established [6, 14].

The transcriptional control that directs the generation of NKp46^+^ ILC3 from NKp46^−^ precursors has not been fully elucidated, however, signals through the aryl hydrocarbon receptor (Ahr) in CCR6^−^ILC3s drives their expansion after birth, and Notch signals in conjunction with the microbiota lead to upregulation of T-bet, acquisition of NKp46 expression and IFN-γ secretion [6, 9].

We have observed that Zbtb1 was required for upregulation of T-bet leading to induction of NKp46 and acquisition of IFN-γ secretion, however, our data does not support a direct function of Zbtb1 in regulating T-bet expression, as ILC1 in siLP are present in normal numbers and have normal T-bet levels. It is also possible that Zbtb1 controls the response to proinflammatory cytokines, such as IL-12 and IL-18, which drive T-bet expression in ILC3s, leading to repression of Rorγt and conversion into ILC1-like cells (exILC3) [9]. Further studies are required to elucidate if there is a deficiency of exILC3 cells in ScanT mice or the mechanisms by which Zbtb1 regulates the generation of NKp46^+^ILC3 cells.

Rag-deficient mice lacking T and B cells have a compensatory expansion of ILCs, and CD4 T-cells were shown to negatively regulate the number of IL-22^+^ILC3 cells in the intestinal lamina propria [26]. As ScanT mice lack T-cells, it was unexpected to find normal numbers of ILC1, ILC2 and ILC3 subsets in ScanT mice. In particular, we didn't find any expansion or defect in IL-22^+^ILC3 cells, which may be consequence of a general defect in immune cell development in ScanT mice [24].

*C. rodentium* is a mucosal pathogen of mice that shares several pathogenic mechanisms with *E. coli*, which is a clinically important human gastrointestinal pathogen. ILC3s provide defense against intestinal infections by various pathogenic bacteria, including *C .rodentium* and *C. albicans* fungal infections [27, 28]. ILC3 protective function has been mostly attributed to their ability to produce IL-22 and is most evident in immune compromised mice, underscoring its redundant function with T-cells and other ILC subsets [11]. NKp46^+^ILC3 cells, however exert a non-redundant function in protecting inflammation in the caecum of immunocompetent mice after *C.rodentium* infections [12].

As the initial innate response to *C.rodentium* infections is independent of T-cells [29], we analyzed the pathology after *C.rodentium* infections at very early time points. Interestingly, despite a similar number of ILC3 cells that secrete IL22 in ScanT and wild type mice, we observed a transient increased sensitivity to *C.rodentium* infections in ScanT mice, seen as decreased body weight, increased immune cell infiltration in the intestinal mucosa, shortening of the colons and increased bacteria load in the feces. This phenotype correlated with reduction of IFN-γ^+^ ILC3s, suggesting that a deficiency in IFN-γ and not IL22 may be responsible for these effects. IFN-γ secreting Th1 cells confer a transient protection to *C.rodentium* infections, preventing loss of body weight and leading to an inflammatory response observed as epithelial hyperplasia and ultimately control of the infection [30, 31]. The pathology that we have observed in ScanT mice resembles that of IFN-γ-deficient mice with mild inflammation and poor control of bacterial load, therefore, it is possible that in the absence of T cells, IFN-γ-secreting NKp46^+^ ILC3s play a similar function to that of Th1 CD4 T-cells.

In summary, we have uncovered an unidentified function of Zbtb1 in controlling the generation of NKp46^+^ILC3 cells. Absence of Zbtb1 impaired the generation of IFN-γ^+^ ILC3 cells while it didn't affect the ability of ILC3s to secrete IL22. This phenotype correlated with a transient increased susceptibility to *C.rodentium* infections, suggesting that IFN-γ secretion by ILC3 cells may play a protective function in immunocompromised mice.

## MATERIALS AND METHODS

### Mice

All experiments were approved by the IACUC committee. C57BL/6 and *Rag2*−/−*gc*−/− mice were purchased from Jackson and Taconic labs respectively. ScanT (Zbtb1-mutant) mice were provided by Bruce Beutler and have been previously described [20]. BAC transgenic Zbtb1-GFP reporter mice (ZEG1) were generated by Kangxin Jin and Dr. Derek Sant’Angelo and where previously published [24]. Rorgt^+/GFP^ were described previously and purchased from the Jackson laboratories [32]. Tbx21^−/−^ mice were previously described [32, 33] and were kindly provided Dr. Vanja Lazarevic (NCI, NIH). For the generation of competitive bone marrow chimeras, recipient *Rag2*−/−*gc*−/− of over 2 month of age were sublethally irradiated with one dose of 500 rads before i.v. injection of a mixture of bone marrow cells from donor mice (2-5×10^6^ cells/recipient). Mice were analyzed 2-4 months after reconstitution.

### Infection with *citrobacter rodentium*

*C. Rodentium* infections were performed as previously described [34]. Briefly, strain DBS100 was prepared by selecting a single colony and cultured in LB broth for 8 hours. Mice were inoculated with approximately 1×10^10^ CFU in 200 μL of PBS *via* oral gavage and analyzed at the indicated time points.

### Detection of *citrobacter rodentium*

The DNA was isolated from feces of infected mice by DNeasy kit (Qiagen). The oligonucleotide primer sequences specific for *C. rodentium* *espB* gene are: GCTTCTGCGAAGTCTGTCAA (forward) and CAGTAAAGCGACTTAACAGATT (reverse). Real time PCR was performed in a 10 μl reaction volume with iTaqTM Universal SYBR^®^ Green Supermix (BIO-RAD). Reaction was performed on 7900HT Fast Real-Time PCR System (Applied Biosystems). The relative bacterial load was calculated relative to *β-actin* from host: GAGATTGTCCGTGACATCAAG (forward) and GCTGGAAGAGAGTCTCTGGGC (reverse).

### Histology

Tissues were fixed in 10% neutral buffered formalin (Sigma) and embedded in wax by standard protocols. Hematoxylin eosin staining was performed following standard procedures.

### Cell isolation from the lamina propria and flow cytometry

Cells from small intestinal lamina propria (siLP) and large intestinal lamina propria (LiLP, including caecum and colon) were prepared as previously described [28]. Single-cell suspensions were stained with CD16/32 (2.4G2) and with fluorochrome-conjugated antibodies against any combination of surface antigens. Prior to fixation, Live/Dead Fixable Blue Cell Stain Kit (Invitrogen) was used to exclude dead cells. For intracellular cytokine staining, isolated cells from lamina propria were stimulated for 2 h with phorbol 12-myristate 13-acetate (PMA) (50 ng/ml) and ionomycin (2.5 μg/ml) or IL-23 (5 ng/ml) in the presence of Brefeldin A (1 μg/ml) (Sigma). For examination of transcription factors and intracellular cytokines, cells were subsequently treated with the FOXP3 staining kit (eBioscience) in accordance with the manufacturer's instructions and stained for 20 min at room temperature with fluorochrome-conjugated antibodies. Antibodies specific to mouse CCR6 (140706) and GATA3 (L50-823) were purchased from BD Biosciences; TCR-β (H57), TCR-γδ (GL3), CD11b (M1/70), CD19 (6D5), Gr-1(RB6-8C5), Ly76 (TER-119), CD127 (A7R34), CD45.1 (A20), CD45.2 (104), Sca-1 (E13-161.7), c-Kit (2B8), NKp46 (29A1.4) were purchased from BioLegend; RORγt (B2D), T-bet (4B10), IL-22 (IL22JOP) and IFNγ (XMG1.2) were purchased from eBioscience. Results were analyzed with FlowJo software (Tree Star).

### Cell culture

OP9-DL1 stroma cells were cultured in αMEM containing 10% FBS (vol/vol), 100 U/ml Penicilin, 100 μg/ml Streptomycin, 2.2 g/l Sodium bicarbonate, 50 μM β-mercaptoethanol. NKp46- ILC3s were sorted and seeded on OP9-DL1 stroma cells supplemented with 5 ng/ml Flt3L, 1 ng/ml IL-7, 10 ng/ml SCF and 10 ng/ml IL-2.

### Statistics

Data was analyzed with Prism 6 software (GraphPad) using two-tailed unpaired Student's *t*-test.

## SUPPLEMENTARY MATERIALS FIGURES


