# Supplementary material for: Zbtb1 controls NKp46+ ROR-gamma-T+ innate lymphoid cell (ILC3) development
Source: Oncotarget. 2017 Jul 27;8(34):55877–88. doi: 10.18632/oncotarget.19645 (PMC5593530; doi:10.18632/oncotarget.19645)
Supplement: Supplementary file 1 [file oncotarget-08-55877-s001.pdf]

Zbtb1 controls NKp46$^+$ ROR-gamma-T$^+$ innate lymphoid cell (ILC3) development

SUPPLEMENTARY MATERIALS

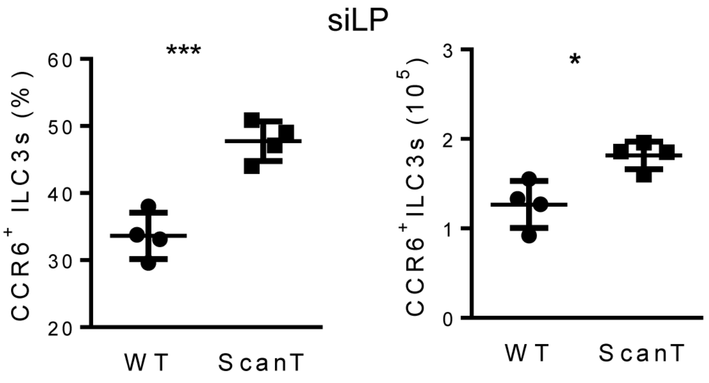

Supplementary Figure 1: Increased CCR6+ILC3 cells in ScanT mice. Percentage and absolute numbers of the indicated ILC3 subsets from the siLP from WT and scanT mice. (All graphs display means ± SD. * $P < 0.05$, *** $P < 0.001$ (two-tailed unpaired Student's t-test).

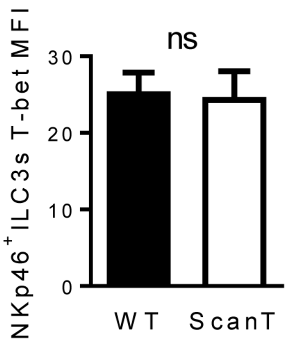

Supplementary Figure 2: Normal T-bet levels in ScanT NKp46+ILC3s. T-bet MFI in NKp46+ILC3s. The graph displays means ± SD. ns $P > 0.05$ (two-tailed unpaired Student's t-test).

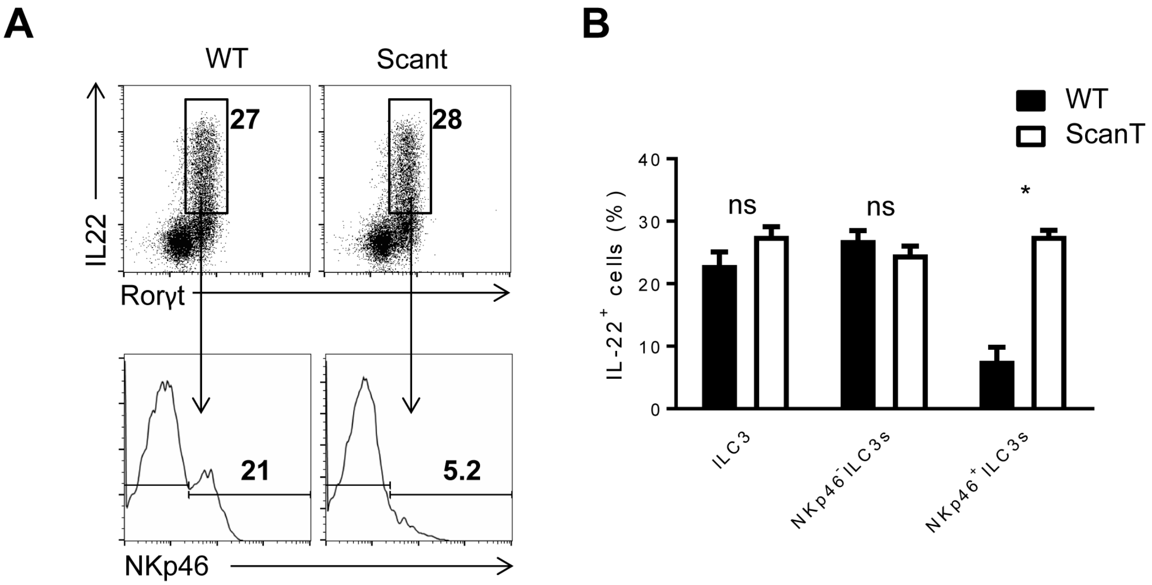

Supplementary Figure 3: Zbtb1-deficiency does not impair IL-22 secretion in total ILC3sin response to IL-23 stimulation. (**A**) FACs analysis of IL-22+ILC3s in the siLP of the indicated mice that have been stimulated with IL-23 for 4 h. (**B**) Statistic results of (A). Results are representative of at least three independent experiments. Numbers adjacent to outlined areas indicate the percentage of events within the gate. * $P < 0.05$, and ns $P > 0.05$ (two-tailed unpaired Student's t-test).

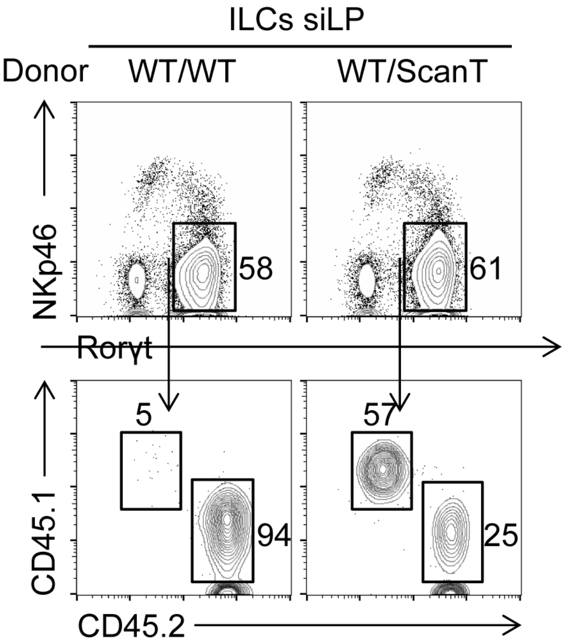

Supplementary Figure 4: Impaired reconstitution of scanT-derived ILC3 cells in competitive bone marrow chimeras FACs analysis of Lin-CD127+ Rorgt+ NKp46-ILC cells in siLP from the same chimera of Figure 3A. Results are representative of at least three independent experiments with three mice in each experimental group. Numbers adjacent to outlined areas indicate the percentage of events within the gate.
